# Associations between reasons for vaping and current vaping and smoking status: Evidence from a UK based cohort

**DOI:** 10.1016/j.drugalcdep.2020.108362

**Published:** 2020-12-01

**Authors:** Jasmine N. Khouja, Amy E. Taylor, Marcus R. Munafò

**Affiliations:** a MRC Integrative Epidemiology Unit at the University of Bristol, United Kingdom; b Bristol Medical School: Population Health Sciences, University of Bristol, United Kingdom; c School of Psychological Science, University of Bristol, United Kingdom; d NIHR Biomedical Research Centre at the University Hospitals Bristol NHS Foundation Trust and the University of Bristol, United Kingdom

**Keywords:** ALSPAC, E-cigarettes, Smoking

## Abstract

**Background:**

This study aimed to discover which young adults vape, the reasons given for vaping, and which reasons for vaping are associated with continued vaping/smoking.

**Methods:**

In a UK cohort of 3,994 young adults, we explored the association of retrospectively-recalled reasons for vaping by 23 years (collected between 2015 and 2016) with vaping/smoking status at 24 years (collected between 2016 and 2017). Using logistic regression, we assessed the association with vaping behaviour among ever vapers who had ever smoked (n = 668), and with smoking behaviour among individuals who regularly smoked prior to vaping (n = 412).

**Results:**

Vaping to quit smoking was associated with higher likelihood of vaping (odds ratio [OR] = 3.51, 95 % confidence interval [95 % CI] = 2.29–5.38), but lower likelihood of smoking at 24 years (OR = 0.50, 95 %CI = 0.32 to 0.78). Vaping to cut down smoking was associated with higher likelihood of vaping (OR = 2.90, 95 % CI = 1.87–4.50) and smoking at 24 years (OR = 1.62, 95 % CI = 1.02–2.58). Vaping out of curiosity was associated with lower likelihood of vaping at 24 years (OR = 0.41, 95 %CI = 0.26 to 0.63) but higher likelihood of smoking at 24 years (OR = 1.66, 95 % CI = 1.04–2.65).

**Conclusions:**

Intention to quit appears important for young adults to stop smoking using e-cigarettes. Public health strategies that encourage vaping specifically for smoking cessation may encourage quitting among young adults.

## Highlights

• Reasons for vaping are associated with vaping and smoking status in UK young adults.
• Vaping out of curiosity and to quit smoking is popular in this population.
• Vaping out of curiosity is associated with discontinued vaping.
• Vaping to stop smoking is associated with quitting smoking.
• Vaping to cut down smoking does not appear to encourage quitting.

## 1 Introduction

Evidence suggests e-cigarettes can aid smoking cessation (Hajek et al., 2019), but some smokers have not tried e-cigarettes, and not all who have tried them have quit smoking (Hartmann‐Boyce et al., 2016; Zhu et al., 2017). It is important to know which individuals vape, why, and whether different reasons for vaping are associated with continued vaping and smoking cessation.

In the US, vapers are more likely to be male and have a lower income (Levy et al., 2017), and in the UK, current and non-current vapers differ in socio-economic status and past year quit attempts (Brown et al., 2014). However, there is limited research exploring differences among young adults in the UK.

Among adults (18+ years) in Great Britain, the primary reasons for vaping relate to smoking cessation (Action on Smoking and Health, 2019). However, evidence from the US suggests that young adults vape primarily out of curiosity (Kong et al., 2015) or because their friends/family vape (Tsai et al., 2018), and Yong et al. (2019) found that they are more motivated to regularly vape for reasons other than quitting smoking. Although current evidence suggests that reasons for vaping are associated with continuation/discontinuation of vaping and smoking (Bold et al., 2016; Nicksic et al., 2019; Saddleson et al., 2016; Yong et al., 2019), there is limited evidence from young adults in the UK.

To inform public health strategies, we aimed to explore the characteristics of young adults who currently or have previously vaped to identify which individuals are more likely to vape. We also sought to investigate whether different retrospectively-recalled reasons for vaping by 23 years are associated with continued vaping and smoking among a UK cohort of young adults.

## 2 Methods

### 2.1 Study population

Young adults enrolled in the Avon Longitudinal Study of Parents and Children (ALSPAC) formed the study sample. The cohort profile and enrolment phases have previously been described (Boyd et al., 2013; Fraser et al., 2013; Northstone et al., 2019). The study website contains details of all the available data (http://www.bristol.ac.uk/alspac/researchers/our-data/). Questionnaires on vaping and smoking were completed at 23 years of age by 4,222 young adults, 3,241 of whom completed questionnaires at 24 years. Supplemental Figure 1 displays the recruitment process and dates of data collection.

Ethics approval for the study was obtained from the ALSPAC Ethics and Law Committee and the Local Research Ethics Committees. Informed consent for the use of data collected via questionnaires and clinics was obtained from participants following the recommendations of the ALSPAC Ethics and Law Committee at the time.

### 2.2 Measures

The Supplementary Material contains detailed information on the measures collected.

#### 2.2.1 Participant characteristics

Participant characteristics included: maternal smoking, body mass index (BMI), risk-taking, education/employment, parenthood status, and mental health.

#### 2.2.2 Exposure

Ever vapers and ever smokers were identified by self-report at 23 years (including those who regularly smoked just prior to vaping). In a multiple-choice question at 23 years, respondents who had ever vaped before were asked “what are/were your reasons for using electronic cigarettes/vaping devices?” and instructed to cross all options that applied: “To help me quit smoking”, “To help me cut down on the number of cigarettes I smoke”, “To help me with cravings in situations where I cannot smoke e.g. travel, indoors”, “Pleasure”, “Curiosity”, “Friends use them”.

#### 2.2.3 Outcome

At 24 years, current vaping and smoking status were self-reported in response to the questions “Do you currently use/vape e-cigarettes or other vaping devices?” and “Have you smoked any cigarettes in the past 30 days?”.

#### 2.2.4 Covariates

Covariates included sex, parental socioeconomic position [SEP], ethnicity and age, which may impact the likelihood of vaping/smoking (Hiscock et al., 2012; Jamal et al., 2016).

### 2.3 Statistical analysis

#### 2.3.1 Differences in participant characteristics

Differences in participant characteristics were assessed using a χ^2^ test or *t*-test to compare ever vapers (vaped at least once) versus never vapers, and former vapers (vaped at least once but self-reported not currently vaping at 23 years) versus current vapers.

#### 2.3.2 Reasons for e-cigarette use and smoking/vaping behaviour

Using logistic regressions, we explored the association between retrospectively-reported reasons for vaping by 23 years and vaping and smoking continuation at 24 years. The six reasons for vaping were analysed individually as binary variables (indicated/not indicated as a reason for use). Vaping and smoking status were treated as binary variables (i.e., current vaper versus non-current vaper, smoker versus non-current smoker). We explored the association between reasons for vaping by 23 years and current vaping at 24 (among ever vapers and ever smokers at 23 years), then explored the association between reasons for vaping by 23 years and current smoking at 24 (among ever vapers at 23 who were regular smokers just prior to first e-cigarette use) with and without adjustment for potential confounders.

#### 2.3.3 Multiple imputation

62 % of respondents who had ever vaped and ever smoked had complete covariate data. We used multiple imputation to increase the sample size available for analysis, minimising bias due to attrition (details in Supplementary Material and Supplemental Table 1). Missing covariate information was imputed for 668 young adults who completed the questionnaires at 23 and 24 years and had ever smoked and ever vaped.

#### 2.3.4 Minimum detectable effect

We had sufficient power (90 %) to detect a minimum odds ratio of 1.51 in a logistic regression of vaping to quit smoking by 23 years and continued vaping at 24 years (Supplementary Material).

## 3 Results

### 3.1 Characteristics of vapers at age 23

The participant characteristics are described in Table 1 and the Supplementary Material, grouped into never vapers (n = 3,013), and ever vapers (n = 981); ever vapers were also grouped into former vapers (n = 814) and current vapers (n = 167). Vapers were more likely to have lower parental SEP at birth, and report risk-taking behaviours and poorer mental health than never vapers.

**Table 1:** Characteristics of the study population for never, former and current e-cigarette users at 23 years (N = 3,994).

				Ever vapers		
	Never vapers (n = 3,013)	Ever vapers (n = 981)	*p*-value	Former vapers (n = 814)	Current vapers (n = 167)	*p*-value
Characteristic	N (%*)	N (%*)		N (%*)	N (%*)	
Female	2005 (67 %)	599 (61 %)	.002	502 (62 %)	97 (58 %)	.387
Parental SEP (manual)	1005 (38 %)	372 (43 %)	.007	297 (42 %)	75 (50 %)	.067
Ancestry (non-European)	116 (4%)	33 (4%)	.509	26 (4%)	7 (5%)	.512
Mother smoked in pregnancy	380 (13 %)	205 (22 %)	<.001	165 (22 %)	40 (25 %)	.314
Harmful/hazardous alcohol use	1033 (49 %)	401 (67 %)	<.001	348 (69 %)	53 (55 %)	.008
Cannabis use (ever)	923 (41 %)	505 (80 %)	<.001	424 (80 %)	81 (79 %)	.673
Other drug use (ever)	401 (18 %)	299 (50 %)	<.001	255 (51 %)	44 (46 %)	.354
Gambled (ever)	322 (15 %)	143 (24 %)	<.001	117 (23 %)	26 (26 %)	.563
Anxiety	730 (36 %)	280 (52 %)	<.001	229 (51 %)	51 (62 %)	.052
Low mood	1269 (63 %)	381 (73 %)	<.001	321 (72 %)	60 (74 %)	.742
Overweight/obese BMI	420 (17 %)	150 (19 %)	.087	122 (19 %)	28 (23 %)	.268
Currently unemployed	151 (7 %)	44 (8 %)	.428	37 (8 %)	7 (9 %)	.920
Parenthood status (had a child)	91 (4 %)	32 (6 %)	.142	24 (5 %)	8 (10 %)	.108
Ever smoked by 23 years	1467 (49 %)	932 (95 %)	<.001	767 (94 %)	>162 (>98 %)	.005
Current smoker at 23 years	355 (12 %)	617 (63 %)	<.001	510 (63 %)	107 (64 %)	.716
Weekly smoker at 23 years**	58 (28 %)	111 (39 %)	.012	89 (37 %)	22 (44 %)	.383
Daily smoker at 23 years**	150 (42 %)	335 (54 %)	<.001	277 (54 %)	58 (55 %)	.940
	Mean (SD)	Mean (SD)		Mean (SD)	Mean (SD)	
Age (initial questionnaire)	23.9 (0.5)	23.9 (0.5)	.854	23.9 (0.5)	23.8 (0.5)	.386

* Due to missing data, the percentage of users refers to the number of participant/participants who responded.

** Only current smokers were asked this question. SEP = Socioeconomic position. The highest socioeconomic position of the mother or father was coded as manual vs non-manual occupation. Harmful/hazardous alcohol use was defined as a score of 8 or more on the Alcohol Use Disorders Identification Test (AUDIT). Anxiety was defined as scores of 5 or more on the GAD-7 which indicated mild to severe anxiety. Low mood was defined as feeling downhearted and depressed in the last 4 weeks and was not an indicator of clinical diagnosis. Unemployment status was defined as not currently being employed or engaging in any form of full or part-time education or training. Less than five participants reported current vaping but never smoking by 23 years; the exact number and percentage have been omitted to protect the anonymity of these participants. Differences between never and ever vapers as well as never, former and current vapers were assessed using χ^2^ for binary outcomes and *t*-test for continuous outcomes.

### 3.2 Reasons for vaping

Reasons for vaping by 23 years are shown in Supplemental Table 2. Current vapers were more likely than former vapers to vape for all reasons except ‘out of curiosity’, the most popular reason for vaping (51 %). Most young adults (56 %) selected only one reason for vaping (Supplemental Table 3).

### 3.3 Reasons for vaping by 23 years and associations with continued vaping and smoking at 24 Years

Due to small numbers of never smokers who had tried vaping at 23 years (n = 47), analyses were restricted to ever smokers who had ever vaped. The study sample consisted of 668 young adults, 412 of whom were regular smokers immediately prior to vaping. Supplemental Tables 4 and 5 display vaping characteristics for vapers at 23 years and transitions in smoking and vaping status. Imputed adjusted results are shown in Table 2. Unadjusted and complete case adjusted results (Supplemental Tables 6 and 7) were consistent (all associations in the same direction with a similar magnitude).

**Table 2:** Associations between reasons for vaping by 23 years and continued vaping and continued smoking at 24 years.

	Vaping at 24 years among ever vapers and ever smokers by 23 years (n = 668)				Smoking at 24 years among ever vapers by 23 years and regular smokers just prior to vaping* (n = 412)			
Reason for vaping by 23 years	N (%)	OR	95 % CI	*p*-value	N (%)	OR	95 % CI	*p*-value
To quit smoking	228 (34 %)	3.51	2.29, 5.38	<.001	217 (53 %)	0.50	0.32, 0.78	.002
To cut down	166 (25 %)	2.90	1.87, 4.50	<.001	158 (38 %)	1.62	1.02, 2.58	.041
To curb cravings	75 (11 %)	4.35	2.57, 7.37	<.001	70 (17 %)	0.84	0.47, 1.49	.553
Pleasure	118 (18 %)	3.22	2.01, 5.15	<.001	57 (14 %)	0.88	0.47, 1.65	.685
Curiosity	351 (53 %)	0.41	0.26, 0.63	<.001	158 (38 %)	1.66	1.04, 2.65	.035
Friends used them	153 (23 %)	0.63	0.37, 1.09	.10	71 (17 %)	1.78	0.95, 3.36	.073

The analyses were run on multiply imputed data for individuals who ever smoked and ever vaped. Both analyses adjusted for demographic factors (sex, ethnicity, socioeconomic position, and age in months at 23-year questionnaire). *This analysis was restricted to those who had reported that they smoked regularly just before they started vaping. OR = Odds ratio. Note: OR = Odds ratio.

Vaping to quit smoking by 23 years was associated with higher likelihood of continuing to vape at 24 years (odds ratio [OR] = 3.51, 95 % confidence interval [95 % CI] = 2.29–5.38, *p* < 0.001) and lower likelihood of continuing to smoke at 24 years (OR = 0.50, 95 %CI = 0.32 to 0.78, *p* = 0.002). Vaping to cut down the number of cigarettes smoked was associated with higher likelihood of continuing to vape (OR = 2.90, 95 %CI = 1.87 to 4.50, *p* < 0.001) and continuing to smoke at 24 years (OR = 1.62, 95 % CI = 1.02–2.58, *p* = 0.041). Vaping to curb cravings for cigarettes (OR = 4.35, 95 % CI = 2.57–7.37, *p* < 0.001) and for pleasure (OR = 3.22, 95 % CI = 2.01–5.15, *p* < 0.001) were also associated with higher likelihood of continuing to vape at 24 years. Vaping out of curiosity was associated with lower likelihood of continuing to vape (OR = 0.41, 95 %CI = 0.26 to 0.63) and a higher likelihood of continuing to smoke at 24 years (OR = 1.66, 95 % CI = 1.04–2.65, *p* = 0.035). Vaping because friends vaped was associated with higher likelihood of continuing to smoke at 24 years (OR = 1.78, 95 % CI = 0.95–3.36, *p* = 0.073).

## 4 Discussion

Similar to previous findings (Brown et al., 2014), vapers were more likely to have lower parental SEP at birth, and report risk-taking behaviours and poorer mental health than never vapers – characteristics previously associated with smoking (Hiscock et al., 2012; Jamal et al., 2016; Lai et al., 2000; Minichino et al., 2013). Consistent with previous findings (Levy et al., 2017), vapers were more likely to be ever, weekly or daily smokers at 23 years, and vaping among never smokers was rare.

Vaping to quit smoking was associated with stopping smoking and with continued vaping, supporting previous findings (Bold et al., 2016; Saddleson et al., 2016). Similar to previous findings (Bold et al., 2016) those who vaped to cut down were more likely to continue vaping and smoking (i.e., be dual users, Supplemental Table 8). Intention to quit may therefore be necessary for e-cigarettes to act as a smoking cessation tool. Consistent with previous research (Bold et al., 2016; Kong et al., 2015), curiosity was the most common reason for vaping among young adults. These users were less likely to continue vaping but more likely to continue smoking.

Despite ALSPAC providing rich data, the measures included were limited. Retrospectively-reporting reasons for vaping could lead to recall bias (e.g., unsuccessful quitters may be less likely to report vaping to quit smoking). The reasons for vaping chosen were not exclusive for each young adult due to the multiple-choice format and open questions and qualitative analysis may have uncovered other potential reasons for vaping which were omitted from this analysis. An ‘other’ option was provided to facilitate open answer responses but was rarely selected (4% of the sample) or solely selected (2%) so was excluded from the analysis. Vaping for flavour-related reasons is common in the US (Landry et al., 2019) but ‘flavours’ was not an option in the 23-year questionnaire. ‘Flavours’ was included in the 24-year questionnaire (selected by 17 % of respondents). There was evidence of a positive association with current vaping but no clear evidence of an association with current smoking in cross-sectional analyses (see exploratory analysis in the Supplementary Material). Additionally, including three smoking-related options may have primed a response biased towards smoking cessation.

Although we employed multiple imputation methods and the ALSPAC response rate has remained consistent in the last eight yearly questionnaires (∼4000 respondents), missing data could have introduced selection bias. Evidence suggests that smokers may be less likely to participate in ALSPAC (Taylor et al., 2018); therefore, there may be fewer individuals vaping or vaping for smoking-related reasons than in the full sample.

The timing of this cohort study may impact the generalisability of the findings. Cigarettes were available to this cohort during adolescence, before e-cigarettes became widely available in 2007. In 2007, the study sample were 17 years old and cigarette initiation peaks at 15–16 years (Marcon et al., 2018), so it is likely that these young adults experimented with cigarettes prior to being exposed to e-cigarettes. Only 1% of the young adults (who were not regularly smoking immediately prior) started regularly smoking after vaping. Today, adolescents are exposed to both cigarettes and e-cigarettes (and to newer devices e.g., pods) which may have an impact on their reasons for vaping and their vaping/smoking status. Although it would be interesting to observe the association between reasons for vaping and later vaping and smoking status among those who were never smokers when they initially vaped, we are unable to identify these individuals within this cohort, and there are too few individuals who had vaped but never smoked at 23 to report meaningful analysis.

### 4.1 Conclusions

Intention to quit appears important for young adults to stop smoking using e-cigarettes. Further research is needed to explore the potential implications of these findings using stronger causal inference methods (e.g., randomising young adults to cut down or quit smoking to explore the impact of intention on smoking cessation using e-cigarettes).

## Contributors

All authors (JNK, AER and MRM) contributed to the design of the study and JNK collated and analysed the data. JNK produced the first of the article which was edited and approved by AER and MRM.

## Role of funding source

This work was supported by the Medical Research Council Integrative Epidemiology Unit at the University of Bristol [grant number MC_UU_00011/7]. The UK Medical Research Council and Welcome (grant number 102215/2/13/2) and the 10.13039/501100000883University of Bristol provide core support for ALSPAC. A comprehensive list of grants funding is available on the ALSPAC website (http://www.bristol.ac.uk/alspac/external/documents/grant-acknowledgements.pdf). This work was also supported by 10.13039/501100000289CRUK (grant numbers C18281/A19169, C57854/A22171), Welcome Trust and the UK Medical Research Council (grant number 092731). This publication is the work of the authors and JK, AT and MM will serve as guarantors for the contents of this paper. The funders had no involvement in the study design, data collection, data analysis, the interpretation of data, in the writing of the report or in the decision to submit the article for publication.

## Declaration of Competing Interest

The authors report no declarations of interest.

## References

[bib0005] Action on Smoking and Health (2019). Use of e-cigarettes (vapourisers) Among Adults in Great Britain. https://ash.org.uk/wp-content/uploads/2019/09/Use-of-e-cigarettes-among-adults-2019.pdf.

[bib0010] Bold K.W., Kong G., Cavallo D.A., Camenga D.R., Krishnan-Sarin S. (2016). Reasons for trying E-cigarettes and risk of continued use. Pediatrics.

[bib0015] Boyd A., Golding J., Macleod J., Lawlor D.A., Fraser A., Henderson J. (2013). Cohort Profile: The’ Children of the 90s’-the index offspring of the Avon Longitudinal Study of Parents and Children. Int. J. Epidemiol..

[bib0020] Brown J., West R., Beard E., Michie S., Shahab L., McNeill A. (2014). Prevalence and characteristics of e-cigarette users in Great Britain: findings from a general population survey of smokers. Addict. Behav..

[bib0025] Fraser A., Macdonald-Wallis C., Tilling K., Boyd A., Golding J., Smith G.D. (2013). Cohort Profile: The Avon Longitudinal Study of Parents and Children: ALSPAC mothers cohort. Int. J. Epidemiol..

[bib0030] Hajek P., Phillips-Waller A., Przulj D., Pesola F., Smith K.M., Bisal N. (2019). A randomized trial of E-Cigarettes versus nicotine-replacement therapy. N. Engl. J. Med..

[bib0035] Hartmann‐Boyce J., McRobbie H., Bullen C., Begh R., Stead L.F., Hajek P. (2016). Electronic cigarettes for smoking cessation. Cochrane Database Syst. Rev..

[bib0040] Hiscock R., Bauld L., Amos A., Fidler J.A., Munafo M. (2012). Socioeconomic status and smoking: a review. Addiction Reviews.

[bib0045] Jamal A., King B.A., Neff L.J., Whitmill J., Babb S.D., Graffunder C.M. (2016). Current cigarette smoking among adults - United States, 2005-2015. Mmwr-Morbidity and Mortality Weekly Report.

[bib0050] Kong G., Morean M.E., Cavallo D.A., Camenga D.R., Krishnan-Sarin S. (2015). Reasons for electronic cigarette experimentation and discontinuation among adolescents and young adults. Nicotine Tob. Res..

[bib0055] Lai S.H., Lai H., Page J.B., McCoy C.B. (2000). The association between cigarette smoking and drug abuse in the United States. J. Addict. Dis..

[bib0060] Landry R.L., Groom A.L., Vu T.-H.T., Stokes A.C., Berry K.M., Kesh A. (2019). The role of flavors in vaping initiation and satisfaction among U.S. adults. Addict. Behav..

[bib0065] Levy D.T., Yuan Z., Li Y. (2017). The prevalence and characteristics of E-Cigarette users in the U.S. Int. J. Environ. Res. Public Health.

[bib0070] Marcon A., Pesce G., Calciano L., Bellisario V., Dharmage S.C., Garcia-Aymerich J. (2018). Trends in smoking initiation in Europe over 40 years: A retrospective cohort study. PLoS One.

[bib0075] Minichino A., Bersani F.S., Calo W.K., Spagnoli F., Francesconi M., Vicinanza R. (2013). Smoking behaviour and mental health disorders-mutual influences and implications for therapy. Int. J. Environ. Res. Public Health.

[bib0080] Nicksic N.E., Snell L.M., Barnes A.J. (2019). Reasons to use e-cigarettes among adults and youth in the Population Assessment of Tobacco and Health (PATH) study. Addict. Behav..

[bib0085] Northstone K., Lewcock M., Groom A., Boyd A., Macleod J., Timpson N., Wells N. (2019). The Avon Longitudinal Study of Parents and Children (ALSPAC): an update on the enrolled sample of index children in 2019. Wellcome Open Res..

[bib0090] Saddleson M.L., Kozlowski L.T., Giovino G.A., Goniewicz M.L., Mahoney M.C., Homish G.G., Arora A. (2016). Enjoyment and other reasons for electronic cigarette use: results from college students in New York. Addict. Behav..

[bib0095] Taylor A.E., Jones H.J., Sallis H., Euesden J., Stergiakouli E., Davies N.M. (2018). Exploring the association of genetic factors with participation in the Avon Longitudinal Study of Parents and Children. Int. J. Epidemiol..

[bib0100] Tsai J., Walton K., Coleman B.N., Sharapova S.R., Johnson S.E., Kennedy S.M., Caraballo R.S. (2018). Reasons for electronic cigarette use among middle and high school students - national youth tobacco survey, United States, 2016. Mmwr-Morbidity and Mortality Weekly Report.

[bib0105] Yong H.H., Borland R., Cummings K.M., Gravely S., Thrasher J.F., McNeill A. (2019). Reasons for regular vaping and for its discontinuation among smokers and recent ex-smokers: findings from the 2016 ITC Four Country Smoking and Vaping Survey. Addiction.

[bib0110] Zhu S.H., Zhuang Y.L., Wong S.S., Cummins S.E., Tedeschi G.J. (2017). E-cigarette use and associated changes in population smoking cessation: evidence from US current population surveys. Bmj-Brit. Med. J..

