# Supplementary material for: Associations between reasons for vaping and current vaping and smoking status: Evidence from a UK based cohort
Source: Drug Alcohol Depend. 2020 Dec 1;217:108362. doi: 10.1016/j.drugalcdep.2020.108362 (PMC7768186; doi:10.1016/j.drugalcdep.2020.108362)
Supplement: Supplementary file 2 [file mmc2.docx]

**Supplementary Materials**

**Additional Questionnaire Information**

Study data from 22 years onwards were collected and managed using REDCap electronic data capture tools hosted at the University of Bristol (Harris et al., 2009). The 23+ questionnaire was completed by the young adults between the ages of 23 and 25 and the 24+ questionnaire was completed a year later when the young adults were between 24 and 26 years. We refer to these time points as 23 years and 24 years respectively. The median time between 23- and 24-year questionnaire completion was 12 months. On average participants vaped at least 2 years before questionnaire completion.

**Additional Variable Information**

***Vaping and smoking.*** Vaping and smoking were assessed at 23 and 24 years. To determine whether questionnaire respondents had ever vaped, they were asked “Have you ever used/vaped an electronic cigarette (e-cigarette) or other vaping device?” To determine whether they had ever smoked, they were asked “Have you ever smoked a whole cigarette (including roll-ups)?” To determine whether respondents were regular smokers immediately prior to vaping (i.e., prior smokers), they were asked “Did you smoke tobacco regularly just before you started using electronic cigarettes/vaping devices?” In addition to the reasons for e-cigarette use answer options displayed in the main text, an “Other” option was provided but rarely selected so was not included in this analysis.

***Maternal smoking in pregnancy.*** Maternal smoking in pregnancy was recorded at 18 weeks gestation. Maternal smoking in pregnancy was based on whether the mother responded yes to smoking in the first 3 months of pregnancy.

***Body mass index.*** Body mass index (BMI) was measured in clinic at 17 years. BMI was calculated from self-reports of height and weight to the nearest one decimal place using the calculation: weight(kg)/height(m)^2^.

***Drug use.*** At 20 years, the young people reported whether they had ever smoked a whole cigarette (response options: yes/no). Alcohol use at 20 was measured using the Alcohol Use Disorders Identification Test (Babor, Higgins-Biddle, Saunders, & Monteiro, 2001). Cannabis use was measured via a question asking if the young person had tried cannabis (response options: yes/no). Other drug use was determined by a response of one or more when asked how many other illicit drugs the young person had ever used.

***Gambling.*** Problem gambling at 20 years was indicated using the problem gambling severity index, an abbreviated version of the Canadian Problem Gambling Index (Ferris & Wynne, 2001). Scores were dichotomised as no problematic gambling vs problematic gambling.

***Education/employment.*** Education and employment status were gathered via a question asking the young person if they were currently in education, training or employment at 21 years (response options: yes/no).

***Parenthood status.*** The young people were asked whether they were a parent at 21 years old. Responses of ‘Yes – biological’ and ‘Yes – non-biological’ were recoded to ‘yes’ to create a binary variable (yes/no).

***Mental health.*** Anxiety was measured using the General Anxiety Disorder (GAD-7) questionnaire (Spitzer, Kroenke, Williams, & Lowe, 2006). Low mood was measured as the amount of time in the 4 weeks prior to completing the questionnaire at 21 years that the young person had felt downhearted and depressed. Response options included: ‘All of the time’, ‘Most of the time’, ‘Some of the time’, ‘A little of the time’, and ‘None of the time’. This was recoded into a binary variable of ‘None of the time’ vs all other responses.

***Sex assigned at birth***. Sex was recorded at birth as male or female.

***Parental socioeconomic position.*** Socio-economic position was recorded at 18 weeks gestation and based on the higher of the mother or partner's occupational social class using the 1991 British Office of Population and Census Statistics classification.

***Ethnicity.*** Ethnicity was classified as white or non-white and was recorded at 32 weeks gestation. The young person’s ethnic background was defined as non-white if their mother responded that she or her partner was any other race or ethnic group than white.

**Multiple Imputation**

Of the young adults who completed the questionnaire on vaping at 23 years, 62% stated they had ever vaped, ever smoked and had complete data including all covariates (22% were missing outcome data, 14% were missing covariate data). We used multiple imputation, a recommended method to account for missing data (Sterne et al., 2009), to increase the sample size available for analysis and minimise bias due to attrition. Data was imputed for missing covariate information; all those included in the analysis had complete exposure and outcome data. Adjusted analyses were repeated using multiply imputed data. The multiple imputation by chained equations procedure was completed using the ICE package in Stata 15.1 which created 100 datasets with 20 cycles. Data were imputed for the 668 young adults who completed the questionnaires at 23 and 24 years and responded that they had ever smoked and ever used an e-cigarette.

**Minimum Detectable Effect Calculation**

To calculate the minimum detectable effect, we assumed an alpha threshold of 5% and 90% power for a two-tailed test. The calculation was based on a logistic regression of vaping to quit smoking by 23 years and vaping status (vaping versus non-vaping) at 24 years. The calculation was based on a 10% probability of an event in the control group; 440 young adults had vaped for reasons other than to quit smoking, 45 of these were vapers, and 395 were non-vapers at 24 years.

**Characteristics of Vapers at 23 Years**

A higher percentage of ever vapers were male, of lower parental SEP at birth, had a mother who smoked in pregnancy, engaged in other potentially addictive or harmful behaviours (harmful or hazardous drinking, drug use, gambling), reported anxiety or low mood, and had smoked by the age of 20 or 23 years and were more likely current, weekly or daily smokers than never vapers at 23 years. Vaping among never smokers was rare; 5% of participants who had ever vaped had never smoked, and <1% defined themselves as current vapers who had never smoked at 23 years. On average, participants were 23 years old across all groups at the initial questionnaire. The age at which the young adults first vaped ranged from 17 to 24 years (SD = 1; median = 22). The age at which young adults first vaped was similar among current and former vapers (median = 22 years of age). There were few clear differences between former and current vapers. Current vapers were more likely to have lower parental SEP at birth, report anxiety and have smoked by the age of 23 years but were less likely to be hazardous or harmful alcohol users than former vapers.

**Additional and Exploratory Analysis**

As we cannot determine whether individuals were dual using products in the main analysis, we further explored the association between reasons for vaping and later vaping and smoking status using multinomial logistic regression. Vaping and smoking status was categorised into four groups: current smoker (smoking but not currently vaping), dual user (currently vaping and smoking), current vaper (vaping but not currently smoking), or neither user (not currently vaping nor smoking). Analyses were adjusted for demographic factors (sex, ethnicity, parental SEP, and age). Analyses were restricted to ever vapers who had 1) ever smoked at 23 years and 2) regularly smoked just prior to vaping. The results are displayed in Supplemental Table 8.

In an exploratory analysis (n = 251), we restricted the analysis of vaping out of curiosity to ever vapers and ever smokers who were non-current smokers just prior to vaping. Among those who were not smoking just prior to vaping, vaping out of curiosity was associated with a lower likelihood of current vaping at 24 years (aOR = 0.24, 95% CI 0.10 to 0.61) but was not clearly associated with current smoking at 24 years (aOR = 0.63, 95% CI 0.34 to 1.16). However, restricting the data in this way could lead to collider bias.

Vaping for ‘flavours’ was only included in the 24-year questionnaire. In an exploratory cross-sectional analysis, there was evidence that vaping for flavours by 24 years was associated with increased likelihood of vaping (aOR = 1.70, 95% CI 1.15 to 2.53), but was not clearly associated with smoking (aOR = 1.06, 95% CI 0.59 to 1.95). Analyses were adjusted for sex, parental SEP at birth, ethnicity, and age at the 24-year questionnaire and restricted to ever vapers at 24 years who had ever smoked or smoked just prior to vaping respectively. As this analysis is cross-sectional, limited conclusions can be drawn from this.

**Supplemental Figure 1. Flow chart depicting the process of data inclusion in the analysis of the associations between reasons for vaping at 23 years and vaping and smoking at 24 years.**

15,454 pregnant women recruited with expected delivery dates between 1^st^ April 1991 and 31^st^ December 1992 resulting in 15,589 foetuses

15,589 live born offspring (singletons and twins) who survived to at least age 1 year and have not subsequently withdrawn

4,222 completed the questionnaire containing smoking and e-cigarette use items at 23 years (data collected between November 2015 and September 2016)

115 recruited in Phase IV were excluded 109 did not respond to e-cigarette items 4 triplets/quadruplets were excluded

3,994 were included in the comparison of ever and never e-cigarette users

3,241 also completed the questionnaire containing smoking and e-cigarette use items at 24 years (data collected between November 2016 and December 2017)

668 had exposure and outcome data and had ever smoked and ever vaped at age 23 years*

578 individuals had complete covariate information*

*Figures shown for ever vapers and ever smokers, for ever vapers and prior smokers n = 412 and n = 360 respectively.

**Supplemental Table 1. Details of imputed variables.**

| **Variable** | Type of variable (number of categories if categorical) | Number (%) with missing data | Regression model used to predict missing data in this variable |
| --- | --- | --- | --- |
| **Outcome variable** |  |  |  |
| Use status | Categorical (4) | 0 (0%) | N/A |
| **Exposure variables** |  |  |  |
| To quit | Binary | 0 (0%) | N/A |
| To cut down | Binary | 0 (0%) | N/A |
| To curb cravings | Binary | 0 (0%) | N/A |
| For pleasure | Binary | 0 (0%) | N/A |
| Curiosity | Binary | 0 (0%) | N/A |
| Friends used them | Binary | 0 (0%) | N/A |
| **Covariates** |  |  |  |
| Sex | Binary | 0 (0%) | N/A |
| Parental SEP at birth | Binary | 75 (44%) | Logistic regression |
| Ethnicity | Binary | 62 (9%) | Logistic regression |
| Age at 23+ questionnaire completion | Continuous | 7 (1%) | Linear regression |
| **Auxiliary variables (i.e., variables not included in the analysis model)** |  |  |  |
| Type of device used | Categorical (3) | 0 (0%) | N/A |
| Maternal smoking in pregnancy | Binary | 46 (7%) | Logistic regression |
| BMI | Continuous | 121 (18%) | Linear regression |
| Cannabis use | Binary | 207 (31%) | Logistic regression |
| Other drug use | Binary |  | Logistic regression |
| AUDIT score | Continuous | 222 (33%) | Linear regression |
| Ever smoked at age 20 | Binary | 202 (30%) | Logistic regression |
| Gambling problems | Binary | 228 (34%) | Logistic regression |
| Condom use | Binary | 298 (45%) | Logistic regression |
| Anxiety | Continuous | 256 (38%) | Linear regression |
| Low mood | Binary | 262 (39%) | Logistic regression |
| Employment and education status | Binary | 266 (40%) | Logistic regression |
| Parenthood status | Binary | 251 (38%) | Logistic regression |
| Ever smoked by 17 years | Binary | 189 (28%) | Logistic regression |
| Ever smoked by 18 years | Binary | 308 (46%) | Logistic regression |
| Ever smoked by 20 years | Binary | 202 (30%) | Logistic regression |
| Ever smoked by 21 years | Binary | 259 (39%) | Logistic regression |
| Ever smoked by 22 years | Binary | 143 (21%) | Logistic regression |
| Current smoker at 18 years | Binary | 366 (55%) | Logistic regression |
| Current smoker at 21 years | Binary | 276 (41%) | Logistic regression |
| Current smoker at 22 years | Binary | 143 (21%) | Logistic regression |
| Ever used an e-cigarette by 22 years | Binary | 145 (22%) | Logistic regression |
| Parental SEP at 8 months | Binary | 397 (59%) | Logistic regression |
| Parental SEP at 2 years | Binary | 376 (56%) | Logistic regression |
| Parental SEP at 3 years | Binary | 412 (62%) | Logistic regression |
| Parental SEP at 4 years | Binary | 374 (56%) | Logistic regression |
| Paternal ethnic group | Binary | 94 (14%) | Logistic regression |
| Maternal ethnic group | Binary | 156 (23%) | Logistic regression |
| Paternal ethnic group (reported by mother) | Binary | 210 (31%) | Logistic regression |
| Number of cigarettes mother smoked per day (32 weeks gestation) | Continuous | 106 (16%) | Linear regression |
| Age YP completed 21+ questionnaire | Continuous | 248 (37%) | Linear regression |
| 21+ completion date based on date received | Binary | 248 (37%) | Logistic regression |
| Age YP completed 22+ questionnaire | Continuous | 140 (21%) | Linear regression |
| 22+ completion date based on date received | Binary | 140 (21%) | Logistic regression |
| BMI at 8 | Continuous | 381 (57%) | Linear regression |
| BMI at 16 | Continuous | 312 (47%) | Linear regression |
| YP tried cannabis by 15 years | Binary | 198 (30%) | Logistic regression |
| YP tried cannabis by 16 years | Binary | 187 (28%) | Logistic regression |
| YP tried cannabis by 17 years | Binary | 272 (41%) | Logistic regression |
| YP tried cannabis by 18 years | Binary | 308 (46%) | Logistic regression |
| YP tried cannabis by 22 years | Binary | 145 (22%) | Logistic regression |
| Tried other drugs by 16 since 15 - aerosols | Binary | 200 (30%) | Logistic regression |
| Tried other drugs by 16 since 15 - gas | Binary | 200 (30%) | Logistic regression |
| Tried other drugs by 16 since 15 - glue | Binary | 198 (30%) | Logistic regression |
| Tried other drugs by 16 since 15 - solvents | Binary | 202 (30%) | Logistic regression |
| Tried other drugs by 16 since 15 - poppers | Binary | 191 (29%) | Logistic regression |
| Tried other drugs by 16 since 15 - amphetamines | Binary | 190 (28%) | Logistic regression |
| Tried other drugs by 16 since 15 - ecstasy | Binary | 188 (28%) | Logistic regression |
| Tried other drugs by 16 since 15 - LSD | Binary | 191 (29%) | Logistic regression |
| Tried other drugs by 16 since 15 - magic mushrooms | Binary | 190 (28%) | Logistic regression |
| Tried other drugs by 16 since 15 - cocaine | Binary | 191 (29%) | Logistic regression |
| Tried other drugs by 16 since 15 - crack | Binary | 192 (29%) | Logistic regression |
| Tried other drugs by 16 since 15 - heroin | Binary | 193 (29%) | Logistic regression |
| Tried other drugs by 16 since 15 - ketamine | Binary | 191 (29%) | Logistic regression |
| Tried other drugs by 16 since 15 - steroids | Binary | 192 (29%) | Logistic regression |
| Number of illicit drugs used by 22 years | Binary | 168 (25%) | Logistic regression |
| Ever drank alcohol by 16 years | Binary | 189 (28%) | Logistic regression |
| Ever drank alcohol by 17 years |  | 266 (40%) |  |
| AUDIT score at 17 years | Continuous | 272 (41%)) | Linear regression |
| Ever drank alcohol by 18 years | Binary | 307 (46%) | Logistic regression |
| Ever drank alcohol by 20 years | Binary | 200 (30%) | Logistic regression |
| Ever drank alcohol by 22 years | Binary | 144 (22%) | Logistic regression |
| AUDIT score at 22 years | Binary | 158 (24%) | Logistic regression |
| Alcohol dependence (DSM-4) | Binary | 159 (24%) | Logistic regression |
| Alcohol abuse (DSM-4) | Binary | 152 (23%) | Logistic regression |
| Gambled by 18 years | Binary | 290 (43%) | Logistic regression |
| Had sexual intercourse in last year at 15 years | Binary | 492 (74%) | Logistic regression |
| Used condom when last had sex at 15 years | Binary | 564 (84%) | Logistic regression |
| Had sexual intercourse in last year at 23 years | Binary | 7 (1.05%) | Logistic regression |
| Used condom when last had sex at 23 years | Categorical (3) | 18 (3%) | Multinomial logistic regression |
| Total CIS-r score at 18 years | Continuous | 231 (35%) | Linear regression |
| Anxiety score at 17 years | Continuous | 231 (35%) | Linear regression |
| YP been sad/miserable/tearful | Binary | 192 (29%) | Logistic regression |
| YP been sad/miserable/tearful for 3+ hours regularly | Binary | 504 (75%) | Logistic regression |
| Depression score at 17 years | Continuous | 231 (35%) | Linear regression |
| YP in full time education at 16 years | Binary | 190 (28%) | Logistic regression |
| YP working part time at 16 years | Binary | 186 (28%) | Logistic regression |
| YP working full time at 16 years | Binary | 186 (28%) | Logistic regression |
| YP in full time education or employment at 20 years | Binary | 208 (31%) | Logistic regression |
| YP become a parent by 16 years | Binary | 196 (29%) | Logistic regression |
| YP become a parent by 20 years | Binary | 195 (29%) | Logistic regression |

**Supplemental Table 2. Reasons for e-cigarette use among former and current vapers by 23 years (N = 981).**

|  | Former vapers  (n = 814) | Current vapers  (n = 167) |  | Full sample  (n= 981) |
| --- | --- | --- | --- | --- |
| Reasons for vaping by 23 | N (%) | N (%) | *p*-value | N (%) |
| To quit smoking | 222 (27%) | 113 (68%) | <0.001 | 335 (34%) |
| To cut down number of cigarettes smoked | 161 (20%) | 68 (41%) | <0.001 | 229 (23%) |
| To help with cravings when unable to smoke | 64 (8%) | 41 (25%) | <0.001 | 72 (7%) |
| For pleasure | 119 (15%) | 54 (32%) | <0.001 | 173 (18%) |
| Out of curiosity | 469 (58%) | 32 (19%) | <0.001 | 501 (51%) |
| Because friends used them | 186 (23%) | 22 (13%) | 0.005 | 208 (21%) |

**Supplemental Table 3. Number of reasons for e-cigarette use selected per respondent (N = 981).**

| Number of reasons selected | Frequency (%) |
| --- | --- |
| 0 | 44 (4%) |
| 1 | 554 (56%) |
| 2 | 223 (23%) |
| 3 | 107 (11%) |
| 4 | 39 (4%) |
| 5 | 10 (1%) |
| 6 | <5 (<1%) |

**Supplemental Table 4. Prevalence of e-cigarette use characteristics among the study sample at 23 years (N = 668).**

| E-cigarette use characteristic | N (%) | |
| --- | --- | --- |
| **Current use at 23** | 105 (16%) | |
| Current smoker and vaper (dual user) | 67 (10%) | |
| Current vaper (not current smoker) | 38 (6%) | |
| Currently vaped at least monthly | 97 (15%) | |
| Currently vapes and has vaped for a month or longer | 91 (14%) | |
|  |  |  |
| **Former use by 23** | 563 (84%) | |
| Used to vape at least once a month but not a current user | 195 (29%) | |
| Used an e-cigarette for a month or longer in the past but not a current user | 144 (22%) | |
|  |  | |
| **Device types used by 23** |  | |
| Ever used a 1st generation device | 200 (30%) | |
| Ever used a 2nd generation device | 340 (51%) | |
| Ever used a 3rd generation device | 410 (61%) | |

**Supplementary Table 5. Smoking and vaping status at 24 years among ever smokers who had ever vaped by 23 years and ever smokers by 23 years who were regular smokers just prior to vaping.**

| Smoking status at 24 years | Ever vapers and ever smokers by 23 years (N=668) | Ever vapers by 23 years and regular smokers just prior to vaping (N=412) |
| --- | --- | --- |
| Current smoker | 330 | 244 |
| Dual user | 62 | 51 |
| Current vaper | 47 | 35 |
| Never user | 229 | 82 |

**Supplemental Table 6. Associations between reasons for vaping by 23 years and current vaping at 24 years among ever vapers and ever smokers.**

|  | Unadjusted (n=668) | | |  | Adjusted (n=578) | | |
| --- | --- | --- | --- | --- | --- | --- | --- |
| Reason for vaping by 23 years | OR | 95% CI | *p*-value |  | OR | 95% CI | *p*-value |
| To quit smoking | 3.43 | 2.24, 5.23 | <.001 |  | 3.97 | 2.50, 6.30 | <.001 |
| To cut down | 2.80 | 1.82, 4.31 | <.001 |  | 3.10 | 1.95, 4.93 | <.001 |
| To curb cravings | 4.34 | 2.58, 7.29 | <.001 |  | 4.95 | 2.82, 8.68 | <.001 |
| Pleasure | 3.20 | 2.02, 5.07 | <.001 |  | 3.08 | 1.86, 5.10 | <.001 |
| Curiosity | 0.42 | 0.27, 0.65 | <.001 |  | 0.39 | 0.24, 0.62 | <.001 |
| Friends used them | 0.62 | 0.36, 1.07 | .084 |  | 0.51 | 0.28, 0.94 | .031 |

The analyses were restricted to individuals who ever smoked and ever vaped. Adjusted analyses adjusted for demographic factors (sex, ethnicity, socioeconomic position, and age in months at 23 year questionnaire). OR = Odds ratio. Note: OR = Odds ratio.

**Supplemental Table 7. Associations between reasons for vaping by 23 years and current smoking at 24 years among ever vapers and regular smokers prior to vaping.**

|  | Unadjusted (n=412) | | |  | Adjusted (n=360) | | |
| --- | --- | --- | --- | --- | --- | --- | --- |
| Reason for vaping by 23 years | OR | 95% CI | *p*-value |  | OR | 95% CI | *p*-value |
| To quit smoking | 0.49 | 0.32, 0.77 | .002 |  | 0.48 | 0.30, 0.77 | .003 |
| To cut down | 1.68 | 1.06, 2.65 | .027 |  | 1.61 | 0.98, 2.64 | .058 |
| To curb cravings | 0.91 | 0.52, 1.60 | .744 |  | 0.85 | 0.46, 1.58 | .614 |
| Pleasure | 0.92 | 0.50, 1.70 | .797 |  | 0.75 | 0.39, 1.43 | .378 |
| Curiosity | 1.68 | 1.06, 2.65 | .027 |  | 1.72 | 1.04, 2.84 | .034 |
| Friends used them | 1.76 | 0.94, 3.30 | .077 |  | 1.74 | 0.91, 3.33 | .092 |

The analyses were restricted to individuals who ever smoked and had smoked regularly just before they started vaping. Adjusted analyses adjusted for demographic factors (sex, ethnicity, socioeconomic position, and age in months at 23 year questionnaire). OR = Odds ratio. Note: OR = Odds ratio.

**Supplemental Table 8. Associations between reasons for vaping by 23 years and vaping and smoking status at 24 years among ever smokers and ever vapers.**

| **Reason for e-cigarette use** | Full sample (n=668) | | | |  | Smokers just prior to vaping (n=412) | | | |
| --- | --- | --- | --- | --- | --- | --- | --- | --- | --- |
| Smoking/vaping behaviour | (Yes/No) | aRRR | 95% CI | *p-*value |  | (Yes/No) | aRRR | 95% CI | *p-*value |
| **To quit** | 228/440 |  |  |  |  | 217/195 |  |  |  |
| Current smoker | 114/216 | 1 | (ref) | (ref) |  | 108/136 | 1 | (ref) | (ref) |
| Current dual user | 36/26 | 2.74 | 1.57, 4.78 | <.001 |  | 33/18 | 2.36 | 1.25, 4.44 | .008 |
| Current vaper | 28/19 | 2.82 | 1.50, 5.31 | .001 |  | 27/8 | 4.35 | 1.88, 10.03 | .001 |
| Neither user | 50/179 | 0.53 | 0.36, 0.78 | .001 |  | 49/33 | 1.83 | 1.09, 3.06 | .021 |
| **To cut down** | 166/502 |  |  |  |  | 158/254 |  |  |  |
| Current smoker | 95/235 | 1 | (ref) | (ref) |  | 91/153 | 1 | (ref) | (ref) |
| Current dual user | 35/27 | 3.35 | 1.90, 5.92 | <.001 |  | 32/19 | 2.98 | 1.57, 5.66 | .001 |
| Current vaper | 12/35 | 0.87 | 0.43, 1.76 | .69 |  | 11/24 | 0.81 | 0.38, 1.76 | .60 |
| Neither user | 24/205 | 0.29 | 0.18, 0.47 | <.001 |  | 24/58 | 0.71 | 0.41, 1.24 | .23 |
| **To curb cravings** | 75/593 |  |  |  |  | 70/342 |  |  |  |
| Current smoker | 30/300 | 1 | (ref) | (ref) |  | 28/216 | 1 | (ref) | (ref) |
| Current dual user | 44/18 | 5.66 | 2.94, 10.90 | <.001 |  | 21/216 | 5.58 | 2.77, 11.25 | <.001 |
| Current vaper | 8/39 | 2.00 | 0.84, 4.73 | .12 |  | 7/28 | 2.07 | 0.81, 5.28 | .13 |
| Neither user | 14/215 | 0.70 | 0.37, 1.35 | .29 |  | 14/68 | 1.73 | 0.85, 3.51 | .13 |
| **Pleasure** | 118/550 |  |  |  |  | 57/355 |  |  |  |
| Current smoker | 40/290 | 1 | (ref) | (ref) |  | 23/221 | 1 | (ref) | (ref) |
| Current dual user | 23/39 | 4.27 | 2.29, 7.98 | <.001 |  | 17/34 | 4.83 | 2.28, 10.22 | <.001 |
| Current vaper | 15/32 | 3.35 | 1.64, 6.85 | .001 |  | 6/29 | 2.09 | 0.76, 5.74 | .15 |
| Neither user | 40/189 | 1.51 | 0.93, 2.44 | .094 |  | 11/71 | 1.55 | 0.71, 3.41 | .27 |
| **Curiosity** | 351/317 |  |  |  |  | 158/254 |  |  |  |
| Current smoker | 163/167 | 1 | (ref) | (ref) |  | 101/143 | 1 | (ref) | (ref) |
| Current dual user | 28/34 | 0.78 | 0.45, 1.37 | .39 |  | 22/29 | 1.01 | 0.54, 1.87 | .99 |
| Current vaper | 10/37 | 0.28 | 0.13, 0.59 | <.001 |  | 5/30 | 0.24 | 0.09, 0.64 | .005 |
| Neither user | 150/79 | 1.94 | 1.36, 2.77 | <.001 |  | 30/52 | 0.81 | 0.48, 1.38 | .44 |
| **Friends used them** | 153/515 |  |  |  |  | 71/341 |  |  |  |
| Current smoker | 78/252 | 1 | (ref) | (ref) |  | 50/194 | 1 | (ref) | (ref) |
| Current dual user | 10/52 | 0.61 | 0.30, 1.27 | .19 |  | 7/44 | 0.60 | 0.25, 1.42 | .24 |
| Current vaper | 8/39 | 0.70 | 0.31, 1.57 | .39 |  | 5/30 | 0.64 | 0.24, 1.75 | .39 |
| Neither user | 57/172 | 1.06 | 0.72, 1.58 | .75 |  | 9/73 | 0.47 | 0.22, 1.01 | .052 |

The models were run on multiply imputed data for individuals who ever smoked and ever vaped (Model 3) and individuals who had been regularly smoking prior to vaping (Model 6). Both models adjusted for demographic factors (sex, ethnicity, socioeconomic position, and age in months at 23 year questionnaire). Note: aRRR = adjusted relative risk ratio. Ref = reference category.

**References**

Babor, T. F., Higgins-Biddle, J. C., Saunders, J. B., & Monteiro, M. G. (2001). *Alcohol Use Disorders Identification Test : Guidelines for use in Primary Care* (Second ed.): World Health Organisation.

Ferris, J., & Wynne, H. J. (2001). The Canadian Problem Gambling Index Final Report. In. Ottawa, ON: Canadian Centre on Substance Abuse.

Harris, P. A., Taylor, R., Thielke, R., Payne, J., Gonzalez, N., & Conde, J. G. (2009). Research electronic data capture (REDCap)--a metadata-driven methodology and workflow process for providing translational research informatics support. *J Biomed Inform, 42*(2), 377-381. doi:10.1016/j.jbi.2008.08.010

Spitzer, R. L., Kroenke, K., Williams, J. B. W., & Lowe, B. (2006). A brief measure for assessing generalized anxiety disorder - The GAD-7. *Archives of Internal Medicine, 166*(10), 1092-1097. doi:DOI 10.1001/archinte.166.10.1092

Sterne, J. A., White, I. R., Carlin, J. B., Spratt, M., Royston, P., Kenward, M. G., . . . Carpenter, J. R. (2009). Multiple imputation for missing data in epidemiological and clinical research: potential and pitfalls. *BMJ, 338*, b2393. doi:10.1136/bmj.b2393
